# The Nurses' Co-Operation

**Published:** 1920-12-04

**Authors:** 


### The Nurses' Co-operation.

WE hope all our readers will make a point of studying the letter signed by four nurses' representatives on the Committee of Management of the Nurses' Co-operation which we publish on page 228 of this issue. The main object with which the Nurses' Co-operation was founded thirty years ago was to secure to nurses the full remuneration for their work. The idea was that of a nurse, Miss Mary Belcher; it was warmly taken up by the late Sir Henry Burdett, and through the energy of a small committee and the financial aid of nurses and sympathisers the Co-operation opened its first offices at 8 New Cavendish Street in January 1891 with a staff of thirty nurses and a capital of £500. The minimum fee fixed for its nurses was 21s. a week, but, whatever the fee, the nurse received then, as she receives now, the whole less a commission of 5 per cent. to 7½ per cent.—*i.e.*, 1s. to 1s. 6d. in the £. The career of the Nurses' Co-operation has been one of continued prosperity, and it now numbers 470 nurses on its staff, while it has invested property amounting to £3,000. It owns the leases of the offices in Langham Street and of the Howard de Walden Club, which it has established and conducts as a residential hostel for Co-operation nurses. Its nurses are insured against employers' liability under the Workmen's Compensation Act, they have a Sickness and Benefit Fund, and there is also a Benevolent Fund for members of the nursing staff who may be in continued ill-health or who need assistance. The difference in her life that such a system of co-operation must mean to the private nurse our readers know only too well. As the Nurses' Co-operation was the pioneer so it has been the means of revolutionising the entire supply of private nurses to the public, for the co-operative system thus inaugurated in 1891 has been adopted as the recognised basis by which private nurses can organise their work and make it a lucrative profession.

